# Spectrophotometric and colorimetric determination of gallium (III) with p-aminohippuric acid-functionalized citrate capped gold nanoparticles

**DOI:** 10.3906/kim-2101-2

**Published:** 2021-06-30

**Authors:** Asiye Aslıhan AVAN

**Affiliations:** 1 Department of Chemistry, Faculty of Engineering, İstanbul University-Cerrahpaşa, İstanbul Turkey

**Keywords:** Gallium, gold nanoparticles, *p* -aminohippuric acid, colorimetric sensor, water analysis

## Abstract

A new technique for sensing Ga(III) concentration based on polyvinyl alcohol-citrate capped gold nanoparticle– *p-* aminohippuric acid hybrid (or three-layer core-shell configurations) has been demonstrated. The *p-* aminohippuric acid capped citrate-gold nanoparticles were comfortably agglomerated in the presence of Ga(III), and the color of the reaction quickly turned from red to violet or blue. Under the detection conditions, a good linear relationship was ideally obtained between the ratio of the absorbance intensity at 620 nm to that at 520 nm (A_620_/A_520_). The linear response range, the detection, and quantification limit was 34.9–418.3 μg/L and 7.6 μg/L, and 25 μg/L, respectively. To reflect the accuracy, the developed sensing approach was evaluated against certified reference materials (TMDA 51.3 fortified water and TMDA 28.3 fortified water). This colorimetric strategy was displayed excellent selectivity for Ga(III) over other examined ions. Additionally, the colorimetric method was properly used to detect the concentrations of Ga in tap water and certified reference material samples with recoveries ranging from 95.4 to 102.0%, displaying that the colorimetric procedure could be safely used for a realistic application.

## 1. Introduction

Gallium (III) is usually accepted as a rare element. Metallic Ga is used mainly in the biomedicine, semiconductor, and electronics industry. In the meanwhile, the risks of Ga in contamination tend to be raised consequently as the spreading of them into the environment is seriously increasing due to the continuous growth of requirements for Ga consumption [1,2]. Moreover, severe damages will be performed to human health if the daily intake amount of Ga(NO_3_)_4_ and GaAs is received at a high level, in particular for the kidneys and hematopoietic system [3,4]. Hence, it performs considerable sense to observe Ga(III) quantity for the estimation of water quality or standard, specifically for the industrial waste discharges. Relatively few papers have been written on this subject in the past 15 years.

The detection of Ga(III) has been accomplished by a variety of instrumental techniques including AES [5–7], ICP [8,9], AAS [10,11] and electrochemical methods [12,13], which always provide satisfying results for their good accuracy. In comparison, some nanoarchitecture based-methodology such as electrochemical, fluorimetry, and colorimetry processes have been obtained more attention and displayed large potential for use in various environmentally friendly applications. The choosing of convenient techniques consisting of a spectroscopic procedure, as well as a measurement organization, is a key factor in analytical technology. The most considerable choosing standards are analytical sensitivity and selectivity to reach the applicability of the method. Other crucial factors, such as costs and complexity of the instrument, have to be evaluated for the ideal and efficient implementation in the industry [14]. The gold nanoparticles (AuNPs) based colorimetric (Col) strategy is a prime selection, which can resolve some inconveniences, such as the time-consuming, relied on the spacious instrument and demanded pretreatment. Owing to the enormous molar extinction coefficient and the relation between the visual attributes and AuNPs sizes, most AuNPs-based probes are employed in Col modes [15]. One of the implementations of these type sensors, based on the color changes generated by the AuNPs agglomeration, is to detect heavy metal ions [16,17]. AuNPs and AgNPs have been particularly concerned in the detection of biomolecules and some metal species due to various crucial superiorities such as without desiring versatile materials and crazy instruments, reading out with the naked eyes, and elevated stability [16–19]. The surface of citrate (Cit) capped AuNPs has the ability of interaction with any metal ions (divalent and trivalent ions), for this reason, to utilize AuNPs for a selective application, the surface functionalization of AuNPs is extremely important. Moreover, it has been demonstrated that AuNPs can be easily functionalized using different kind of ligands (chelating agents), polymers, biomolecules, etc. depending on the application [20]. To attain high selectivity, the surface of AuNPs must be exposed to modification with recognition agents that react selectively with the goal metal ion. For example, AuNPs have been widely exploited as Col sensors for the accurate determination of low levels of toxic metal ions such as As, Cd, Co, Ni, Pb, Cr and so on [21]. Functionalization of AuNPs improves the sensitivity and selectivity of the Col probe. In this context, AuNPs can be functionalized easily with organic molecules that contain thiols, amines, or even phosphine moieties [22]. Chelating agents are chemicals able to form a specific complex with certain metal ions. Chelating agents can be utilized to functionalize AuNPs for the AuNPs to be responsive to specific metal ions. To date, various ligands were used for surface functionalization. There are three crucial protocols of fabricating functionalized AuNPs: (1) physical adsorption (hydrophobic or electrostatic interaction), (2) covalent coupling (strong Au-S bonding), and (3) specific recognition (AuNPs labeled with antibodies) [20,21,23–25]. In recent years, the functionalization of the Cit-AuNPs has been performed with different chelating agents, such as congo red [26], 2,6-dimercaptopurine [27], *p* -aminohippuric acid [28,29], dithizone [30], 2,6-pyridine dicarboxylic acid (PDCA) [31], 1,5-diphenylcarbazide [32] and so on. *p* -aminohippuric acid (or N-(4-Aminobenzoyl) glycine) (PAH) is a biologically respectable compound. PAH is an amide derivative of amino acid glycine and hippuric acid. In 2015 Jin’s group fabricated selective and quantification of trace Cr (III) in water by utilizing PAH-modified Cit-AuNPs [28]. The PAH molecule was attached to the surface of AuNPs by the amine groups in the side chain of PAH at the N-terminal. PAH as a chelating agent following their modifying onto AuNPs pioneers to the high improvement of the selectivity and sensitivity [28]. 

To date, the Col determination of Ga(III) with AuNPs-based probe by UV-vis absorption spectroscopy has not been reported. AuNPs are a crucial candidate for Ga sensing due to their agglomeration attributes together with their excellent optical attributes. Herein, polyvinyl alcohol-citrate capped AuNPs (PVA-Cit-AuNPs) hybrid was confidently employed as a Col sensing material for Ga and further developed a UV-vis spectrophotometric Ga sensor. The citrate-capped AuNPs prepared are stabilized or dispersed in the PVA matrix to form highly stable PVA-Cit-AuNPs.

## 2. Materials and methods

### 2.1. Materials

For all of the experiment steps, distilled water was employed. The following chemicals were utilized in this investigation: trisodium citrate (Trisodium 2-hydroxypropane-1,2,3-tricarboxylate), poly (vinyl alcohol), HAuCl_4_.3H_2_O, *p* -aminohippuric acid, Ga(NO_3_)_3_.9H_2_O, glycine were obtained from Merck (Darmstadt, Germany). Various concentrations (34.9–418.3 μg/L) of Ga(III) were carefully prepared by serial dilutions of Ga(NO_3_)_3_.9H_2_O stock solution to reach the lowest concentration. The *p* -aminohippuric acid (or N-(4-Amino benzoyl) glycine) solution (1.0 × 10^–4^ M) was meticulously prepared by dissolving the convenient quantity of PAH in 8.0 × 10^–3^ M NaOH. Glycine-HCl buffer (Gly-HCl) (0.1 M, pH 2.2–3.6) was exactly prepared by mixing 0.2 M Gly with 0.2 M HCl diluted to make up a total volume of 100 mL. All the metal salts used in the experiments were of analytical grade and dissolved in 0.01M HCl.

### 2.1. Instruments

The following instruments were properly employed in this examination:  All UV-vis spectrophotometric implementations were estimated with a Varian (Cary 100 Bio) UV–vis spectrophotometer. Absorbance densities were measured in a quartz cuvette with light path 10 mm. AuNPs were isolated by centrifugation with Hettich-Universal 320R centrifuge (speed range 500–15,000 rpm) (Hettich, Germany). The pH measurements were carried out using a HANNA HI 221 pH meter (Woonsocket, RI-USA). A heating magnetic stirrer (Daihan Wisd MSH-20A, Soul, Korea) was used to synthesis of AuNPs. Elmasonic E 30H (Singen, Germany) model ultrasonic bath was used for the preparation of solutions. Composites were characterized by scanning electron microscopy (Quanta 450 FEG *-* EDS Field Emission Scanning Electron microscope, FEI Company *,* USA). All infrared spectra were recorded using the Agilent Cary 630 FT-IR analyzer. The utilized laboratory glasswares were fully cleaned in a bath of HCl : HNO_3_ (3 : 1) and then rinsed 3–4 times with distilled water and ultimately dried in the oven at 100 °C before use.

### 2.2. Preparation of PVA-Cit-AuNPs

The synthesis of AuNPs was carried out using a citrate reduction method known as the Turkevich method [33]. Briefly, 50 mL of 8.5 × 10^–4^ M HAuCl_4_ was appropriately heated to reflux with intensely stirring in a round-bottom flask combined with a reflux condenser, and, following this, 5 mL of 1.0% sodium citrate (w/v) was attentively and rapidly incorporated. The mixture obtained was kept boiling for 30 min during which its color converted from yellow to wine red. Ultimately, the solution was cooled in favourable circumstances with continual stirring. Subsequently, to eliminate the excess Cit ions, the resulting Cit-AuNPs were minutely centrifuged at 12,000 rpm for 10 min to deport the free Cit ions and the precipitated AuNPs redispersed in distilled water (50 mL) containing 5.0 mg PVA and the mixture was agitated for 30 min. In this system, PVA was extra added to improve stabilization of the Cit-capped AuNPs against the high salt conditions. The PVA encapsulated Cit-AuNPs demonstrated the surface plasmon resonance (SPR) band at 520 nm *.* A molar extinction coefficient (ε) for Cit-AuNPs and PVA *-* Cit *-* AuNPs was calculated to be ε = 1.23 × 10^8^ mol^–1^ L cm^–1^ at 520 nm. This result obtained is in good agreement with a declared value (ε = 2.78 × 10^8^ mol^–1^ L cm^–1^) [27] *.* Following that, the obtained core–shell nanostructure (PVA *-* Cit *-* AuNPs) was stored at 4 °C for the next experiments. At this time, the color of the double core-shell PVA-Cit-AuNPs was stable over a long time and these results exhibiting that the Cit-AuNPs do not tend to agglomerate in PVA gel. Further, the long-term stability of PVA-Cit-AuNPs was monitored. During 1 month, the absorbance density was monitored at 520 nm and the absorbance density remains the same (RSD = 2.9%) in the inspected period. Consequently, the PVA-Cit-AuNPs solution is quite stable over a long period and hence, the hybrid material obtained can be ideally used in Col sensing methodology.

### 2.3.Preparation of PAH-PVA-Cit-AuNPs 

**Figure 1:**
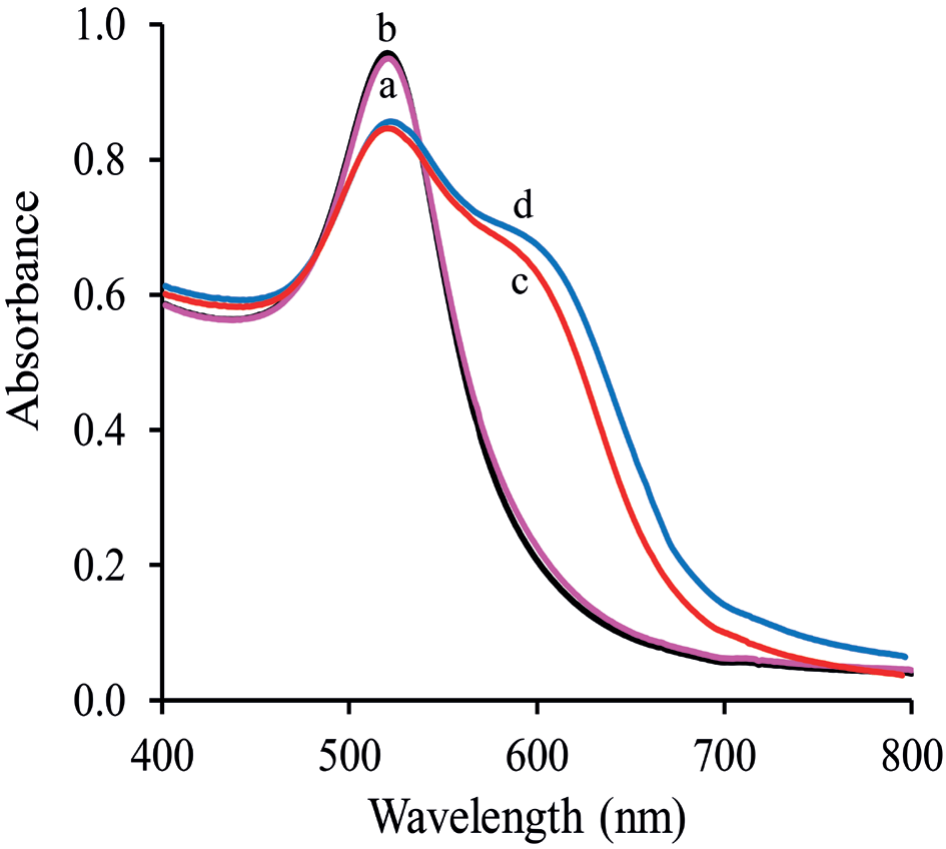
The UV-visible absorption spectrum of PVA-Cit-AuNPs in the absence (a) and presence (b) of Ga(III) and the UV-visible absorption spectrum of PAH-PVA-Cit-AuNPs in the absence (c) and presence (d) of Ga(III), [Ga]: 209.2 μg/L.

The visible surface of two-layer core-shell PVA-Cit-AuNPs can direct interaction with any heavy metal ions. Therefore, the two-layer core-shell PVA-Cit-AuNPs probe is unselective for metal species. To properly use AuNPs for a selective application, the capacity to modify the visible surface is tremendously important. Furthermore, it has been already demonstrated that AuNPs can be easily functionalized using different kind of ligands (or chelating agents), polymers, biomolecules, etc. depending on the application [34–36]. For this study *,* the PAH reagent was properly employed as a chelating agent for the selective and sensitive detection of Ga(III). By immobilizing PAH on a suitable support (AuNPs), analyses can be conducted more sensitive. AuNPs are generally decorated on amine-terminated layers, through electrostatic interaction. Accordingly, the PAH noticeably for the formation of H bonding added to AuNPs and construct the sensitive Col sensor or probe for sensing of a single metal ion. In this regard, the three-layer core *-* shell morphology (PAH-PVA-Cit-AuNPs) was prepared with the PAH capped specific strategy giving to the previously published method with necessary modification [28]. For the preparation of three-layer core *-* shell morphology, 10 µL of 1.0 × 10^-4^ M PAH was incorporated into 1.0 mL PVA-Cit-AuNPs (two-layer core-shell morphology), and the prepared mixture was approximately equilibrated for 30 min at the room temperature and was used a 0.4-mL aliquot of the solution. However, the PVA-Cit *-* AuNPs and PAH-PVA-Cit-AuNPs are unstable and the color fading over time is virtually inevitable. The PAH-PVA-Cit-AuNPs and classic PVA-Cit-AuNPs architectures exhibited an SPR absorption intensity at 520 nm. The solution of PAH-PVA-Cit-AuNPs (three-layer core-shell) was stable and well dispersed in water. The molar absorptivity for PAH-PVA-Cit-AuNPs was 1.33 × 10^8^ mol^–1^ L cm^–1^ at 520 nm. When Ga (III) ions were added into the PAH-PVA-Cit-AuNPs solution, self-assembly occurred instantly, due to the formation of a chelate complex (Ga-PAH complex). Consequently, the color of the sample solution changed immediately from red to blue. Figure 1 shows the absorption spectra for PVA-Cit-AuNPs (curve a), Ga(III)-PVA-Cit-AuNPs (curve b), PAH-PVA-Cit-AuNPs (curve c), and Ga(III)-PAH-PVA-Cit-AuNPs (curve d) at pH = 2.2 in the wavelength range 400–800 nm against a water blank. Unfortunately, the absorbance height value of Ga(III)-PAH-PVA-Cit-AuNPs was higher than Ga(III)-PVA-Cit-AuNPs as can be illustrated in Figure 1. Therefore, the as-prepared three-layer core *-* shell morphology (PAH-PVA-Cit-AuNPs) was chosen and used as a Col sensor to reliably detect Ga(III). For this work *,* a 0.4 mL aliquot of PAH-PVA-Cit-AuNPs solution was ideally employed for the next experiments *.*

### 2.4. Colorimetric detection of Ga(III) 

Colorimetric measuring of Ga(III) was actualized using PAH-PVA-Cit-AuNPs solution. Briefly, 0.4 mL of Ga(III) solution with various concentrations in the range of 34.9–418.3 μg/L and 0.1 mL of 0.1 M Gly-HCl buffer (pH 2.2) are mixed and then diluted to 0.6 mL with distilled water. Subsequently, a 0.4 mL PAH-PVA-Cit-AuNPs probe is carefully added to the solution of interest. To prevent the gathering of AuNPs, the order of additions important. In this concept, the sample volume (final volume) of 1.0 mL was always employed. The mixture prepared was roundly incubated for 5 min at ambient temperature to reach a stable response and the absorbance of the solutions were meticulously measured by UV-vis spectrophotometry or by naked-eyes. The absorption spectra of Ga(III)-PAH-PVA-Cit-AuNPs were recorded in the wavelength range of 400–800 nm. For analytical assessments, the greatness of the absorbance ratio (A_620_/A_520_) was taken into account. For an appreciation of the selectivity, other potential ions (anions and cations) were incorporated and checked under the same experimental conditions.

## 3. Results

### 3.1. SEM analysis

**Figure 2:**
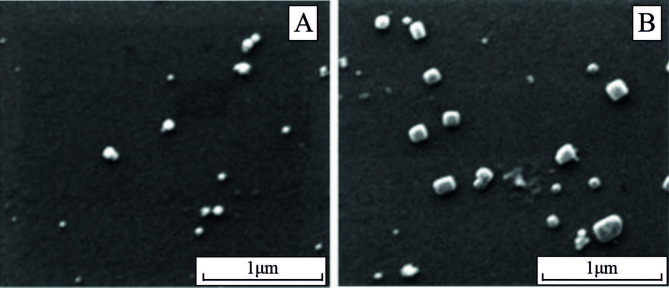
SEM image of PVA-Cit-AuNPs (A) and PAH-PVA-Cit-AuNPs (B).

In the SEM image, the surface morphology of the Cit capped AuNPs was remarkably changed, and the AuNPs sizes were exactly increased after modifying with PAH forming PAH-PVA-Cit-AuNPs as shown in Figure 2A and 2B, respectively. The average diameters of PVA-Cit-AuNPs and PAH-PVA-Cit-AuNPs were found to be 35 ± 8 nm and 96 ± 20 nm. A total of 20 pieces of AuNPs were randomly exploited for diameter calculation from the microscopic field.

### 3.2. FT-IR analysis

**Figure 3:**
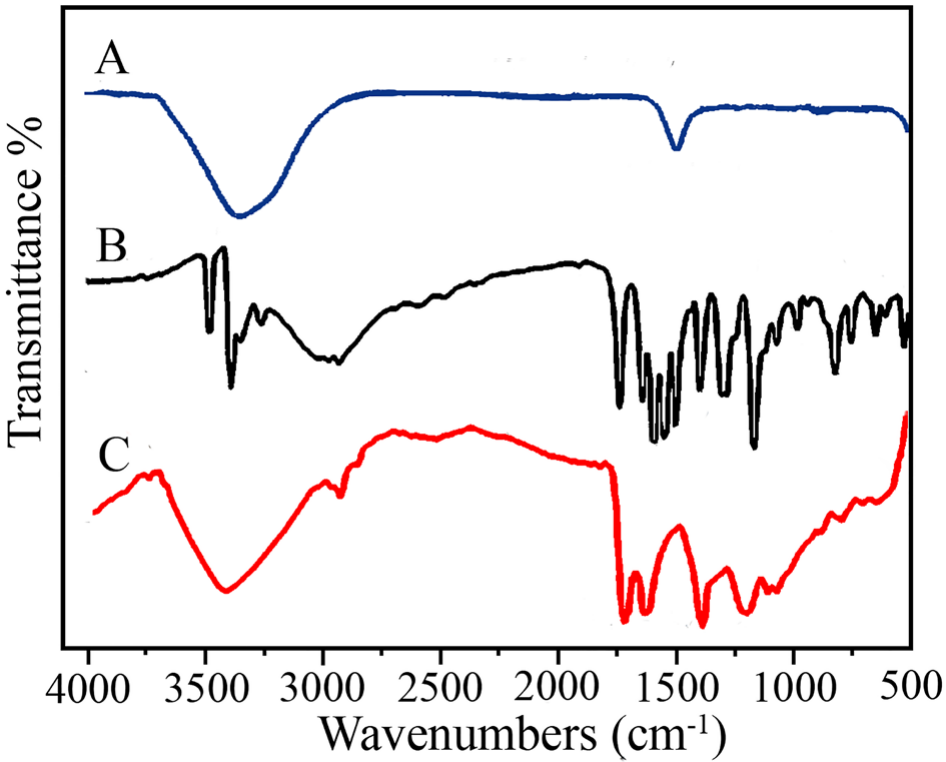
FT-IR spectra of Cit-AuNPs (A), PAH (B) and PAHCit- AuNPs (C).

Figure 3 shows FT-IR spectra of Cit-AuNPs (curve A), PAH (curve B) and PAH-Cit-AuNPs (curve C). The pure PAH has three crucial functional groups: the amide bond, amino group, and a carboxyl group. Spectral characterization of PAH shows several distinct peaks (Figure 3B). The two strong amino group bands obtained at 3374 and 3485 cm^−1^ in FT-IR for symmetric and asymmetric stretching, respectively [37]. For comparison, the FT-IR spectrum of AuNPs shown in Figure 3A. The AuNPs synthesized by the citrate capped method yielded strong bands at 3352 and 1639 cm^–1^. However, when AuNPs functionalized with PAH, the FT-IR spectra of PAH-Cit-AuNPs exhibited additional bands at 1722, 1625 and 1387 and 1200 cm^–1^ which resembled those in the presence of modified onto AuNPs surface. The bands at 2974 cm^−1^ and 2897 cm^−1^ assigned for the C–H stretch. AuNPs should not include any C–H or –OH functional groups, and, thus, the above bands indicated capping of the AuNPs by Cit groups. The band 900–650 cm^–1^ region are due to the N–H out-of-plane bending vibration of the amino group. These characteristic bands (at 3371 cm^−1^, 3462 cm^−1^, and 900–650 cm^–1^)disappeared in the FT-IR spectra of PAH-Cit-AuNPs. Besides, the stretching bands at 1720 сm^−1^ (the C=O stretching band of a carboxyl group), 1400 cm^−1^ (C–N amines) and 1650–1630 cm^−1^ (the C=O stretching bands of the amide) even so existed (Figure 3C). However, the appeared bands merely shifted after the formation of PAH-Cit-AuNPs. FT-IR spectra implicated that the PAH bound onto the Cit-AuNPs via the amino group instead of the amide bond or a carbonyl group.

### 3.3. Colorimetric sensing mechanism of Ga(III)

**Figure 4:**
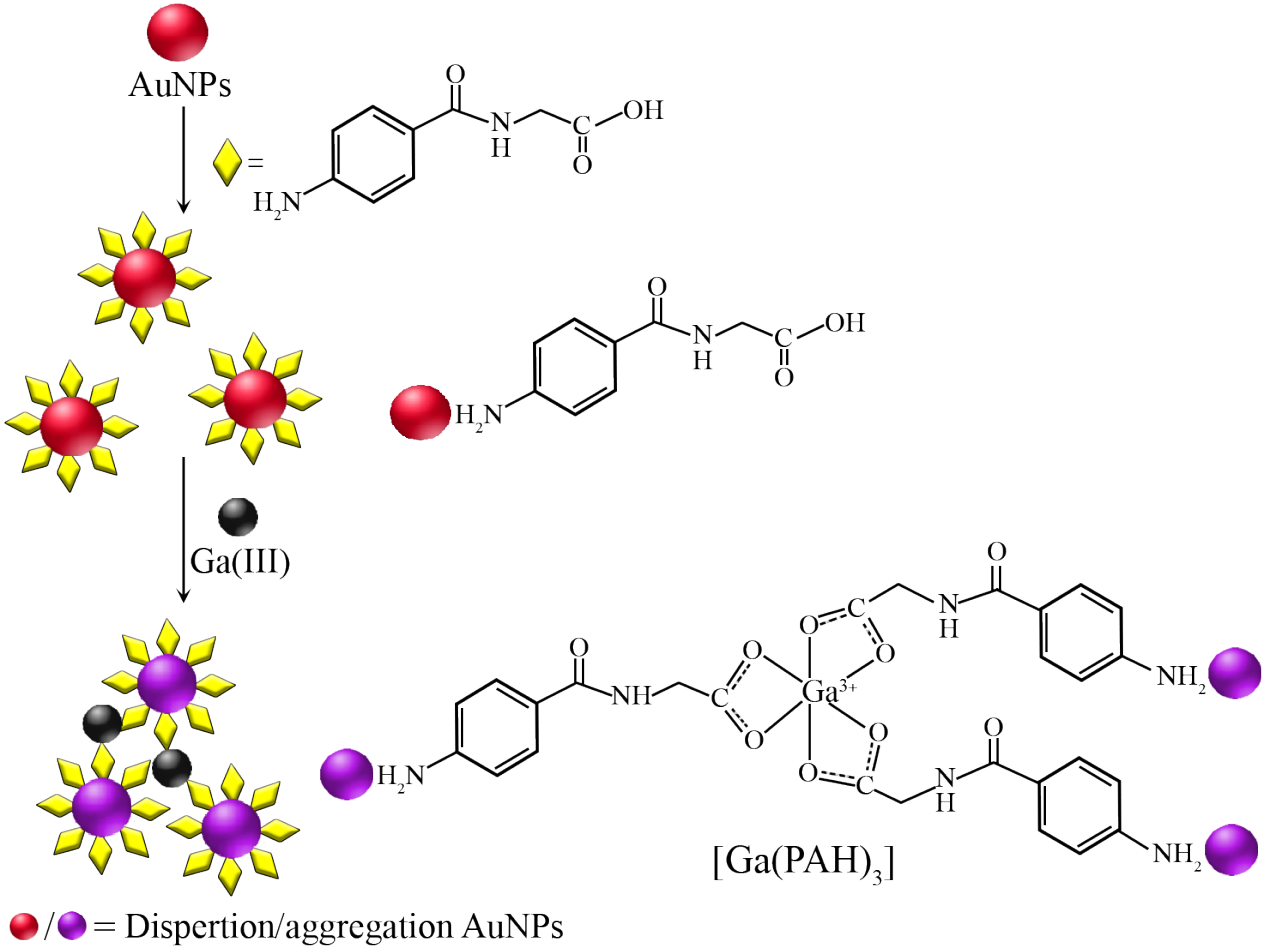
Probably assay mechanism for colorimetric detection of Ga(III) with the PAH-PVA-Cit- AuNPs system.

The color of the solutions is stable over a long period. The results pointed out that the AuNPs prepared do not tend to agglomerate in PVA gel. The crucial role of PVA gel behaves as a holding matrice and in this system, the –OH group of PVA may temporarily connect with the AuNPs through Van der Walls forces. [38]. O’Brien et al. [39] declared that Ga(III) could give a stable Ga(III)-Cit complex with citrate in 1:2 binding stoichiometry (Ga(Cit)_2_^3-^). Also, Ga(III) form complexes with hippuric acid in the ratio of 1:3 [40]. The hippuric acid (HIP) derivatives are coordinated with the trivalent metal ions (such as Fe^3+^, Cr^3+^, In^3+^ and so on) as a bidentate through the carboxylic group [28]. On the other hand, divalent metal ions (M: Zn^2+^, Cd^2+^, Hg^2+^, Cu^2+^ and so on) complexes with PAH and their amine adducts are of the type M(HIP)_2_.H_2_O (formed in the ratio of 1:2) [39,40]. PAH is an N-acylglycine that contains an amine (–NH_2_)group and carboxylic acid (–COOH) group. The probably sensing mechanism is shown in Figure 4. As shown in Figure 4, PAH is linked with Cit-AuNPs through -NH_2_ group to obtain -NH_2_ (PAH) functionalized Cit-AuNPs (Cit-AuNPs-NH_2_) Amine group of PAH can rapidly interact with negatively charged Cit-AuNPs through electrostatic interaction. After the addition of Ga(III), the chelating agent PAH molecule reacts with Ga(III) to form a complex (Ga-PAH complex) through the carboxylic group, and, thus, trigger the agglomeration of PVA-Cit-AuNPs and the color changes were able to be monitored by the naked eyes. Consequently, the three-layer core-shell displays a prominent Col signalling behaviour toward Ga(III) via a visual color change. The inspections indicated that the absorbance intensity of Ga(III)-PAH-PVA-Cit-AuNPs was higher than Ga(III)-PVA-Cit-AuNPs. Therefore, the PAH-PVA-Cit-AuNPs hybrid (three-layer core-shell) can be safely used as a potential probe material for Ga(III) detection. 

### 3.4. Optimization of sensing conditions 

Several related factors such as pH, the PAH concentration and incubation time and the effect of foreign ions on the response were inspected. These crucial factors were evaluated employing 209.2 μg/L Ga(III) due to the absorbance peak profile of Ga(III) (at 620 nm) was much sharper and specific. Further, the absorbance density ratio at 620 nm and 520 nm evaluated (A_620_/A_520_).

#### 3.4.1. Effect of PAH concentration 

**Figure 5:**
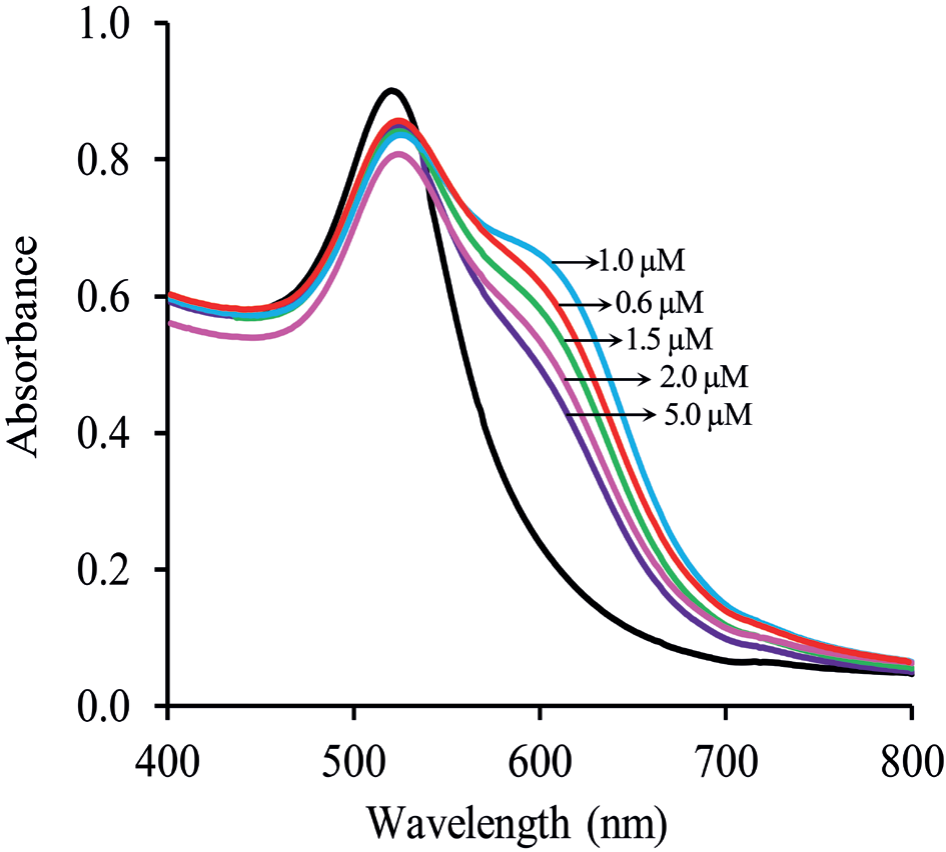
Effect of PAH on the response of the sensor in the presence of 209.2 μg/L Ga(III).

The chelating agent concentration is a crucial element in the UV-vis spectrophotometric inspection. It produces a high absorbance peak when Ga(III) ions added to the solution. The ratio of PAH-PVA-Cit-AuNPs is importance to obtain the best absorbance ratio (A_620_/A_520_). Since the concentration of PAH could effect on PVA-Cit-AuNPs sensing performance, thus, the stoichiometric ratios between PAH and PVA-Cit-AuNPs were favourably investigated. The effect of the PAH concentration on the determination of Ga has been studied. In this context, 1.0 mL PVA-Cit-AuNPs sensor solution was mixed with different concentrations of PAH (in the range of 0.6 to 5 µM) and diluted to 1.050 mL (if desired: the total volume can be ignored) with distilled water and stirred for 30 min at room temperature to obtain PAH-PVA-Cit-AuNPs sensor solutions. A 0.4-mL aliquot of the test sample or blank sample was carefully pipetted into a glass tube. After that, the above procedure was carried out as described in colorimetric detection of Ga(III). The obtained UV–vis spectra were illustrated in Figure 5. A maximum response for Ga(III) has obtained using 0.4 mL of PAH-PVA-Cit-AuNPs sensor solution. Consequently, a 0.4 mL PAH-PVA-Cit-AuNPs solution containing 1.0 µM PAH was employed as a Col probe.

#### 3.4.2. Effect of pH 

**Figure 6:**
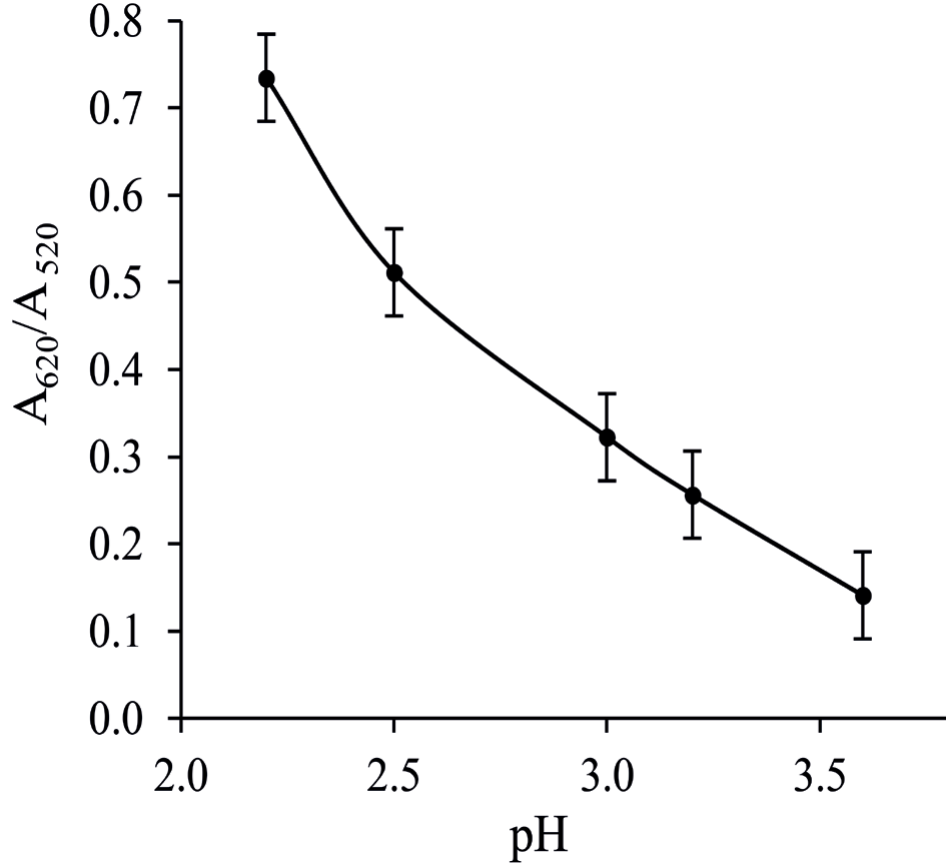
Effect of pH on the response of the Col sensor to the mixture solution containing 209.2 μg/L Ga(III). The error bars represent standard deviation of the mean.

The pH value of the solutions plays a crucial role in the formation of the Ga(III): PAH complex. Thus, the effect of the pH value of the Gly-HCl buffer on the Col response of Ga(III) was inspected in the pH range 2.2–3.6 and the detection of Ga(III) was investigated using UV-vis spectrophotometry. Figure 6 illustrates the effect of pH** on the Col response. As can be observed, the maximum Col performance and highest absorbance peak ratio were obtained at pH 2.2 and meanwhile, the PAH: Ga(III) complex quickly formed. Figure 6 illustrates that the absorbance value decreases from 2.2 to 3.6. Therefore, the ideal pH value of 2.2 was employed for further experiments.

#### 3.4.3. Incubation time

**Figure 7:**
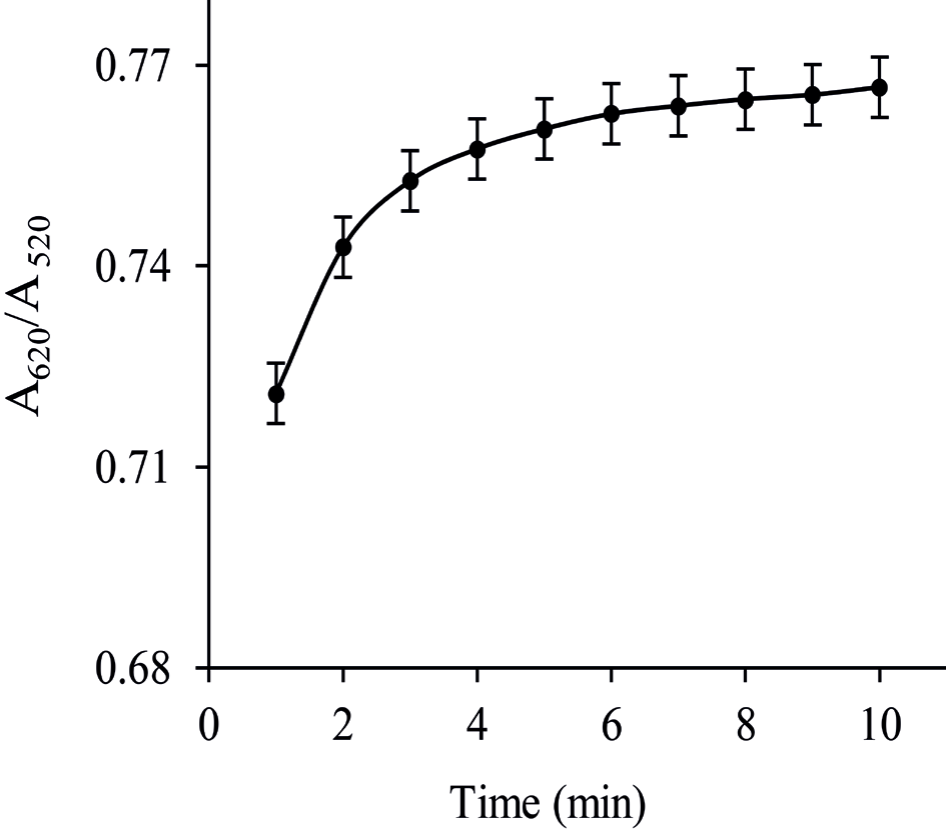
Effect of incubation time on absorbance values of 209.2 μg/L Ga(III). The error bars represent standard deviation of the mean.

To reflect the ideal Col performance, the incubation time carefully optimized as the most valuable influence factor for the sensing. The concentration of Ga(III) ion was 209.2 μg/L. The incubation experiments were carefully investigated at room temperature (25 ± 2 °C). Figure 7 shows the effect of incubation time on the absorbance value (A_620_/A_520_). As shown in Figure 7, the absorbance intensity increased obviously within 5 min and then obtained a platform. Prolonged incubation time did not increase the absorbance value. So, 5 min was favorably employed as the optimal incubation time for remained experiments. 

### 3.5. Sensitivity of the PAH-AuNPs to Ga(III)

**Figure 8:**
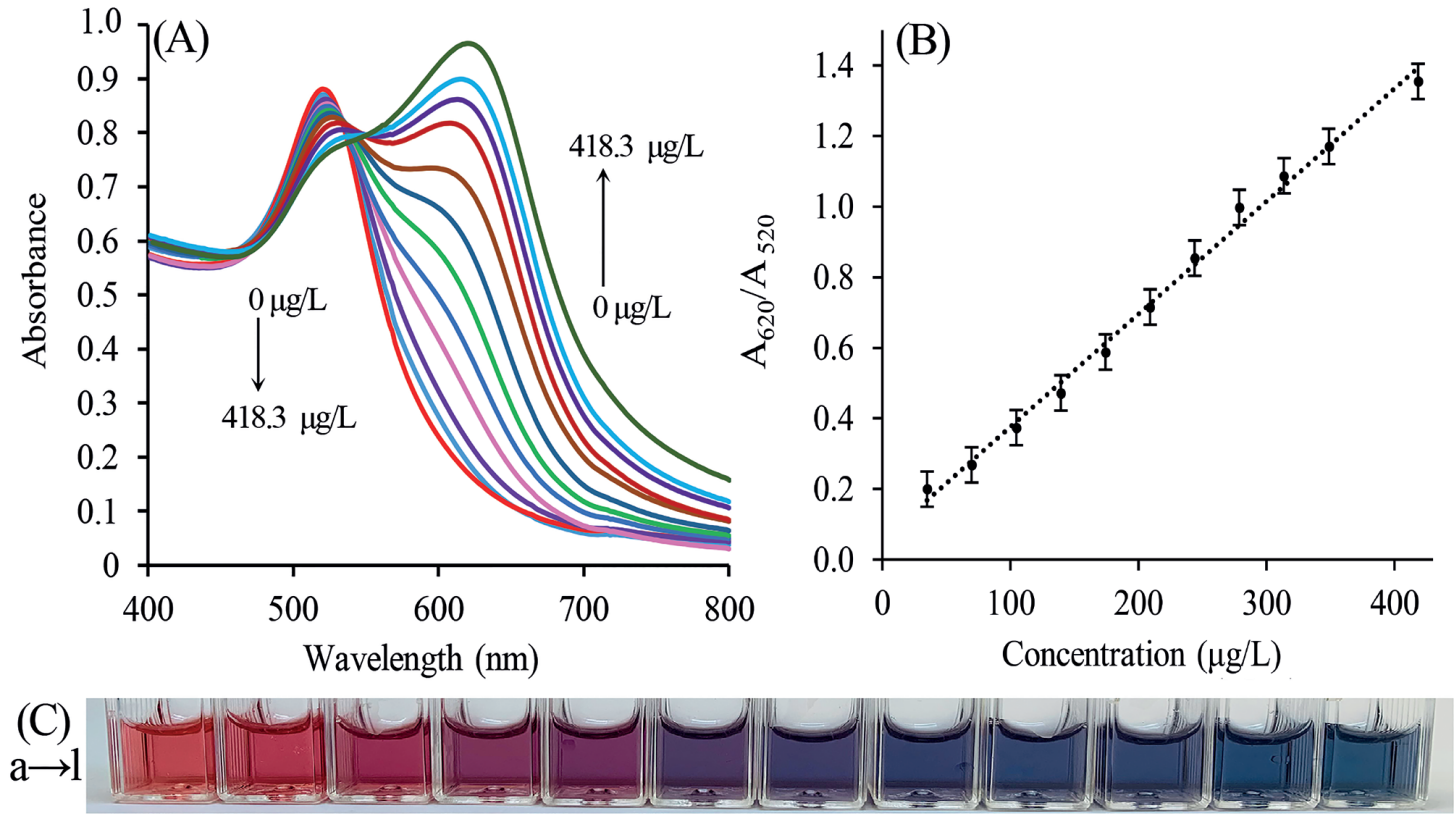
(A) UV–vis spectra of PAH-PVA-Cit-AuNPs as a function of Ga(III) concentrations (from 0 to 418.3 μg/L). (B) The linear calibration curve between absorbance intensity ratio A620/A520 and the concentrations of Ga(III). The errors bars in (B) represent the standard deviation for three independent measurements. (C) Photographs of PAH-PVA-Cit-AuNPs as a function of Ga(III) concentrations.

The color change of sensor (PAH-PVA-Cit-AuNPs) induced by Ga(III) was monitored by UV–vis spectrophotometry. The corresponding UV–vis absorption spectra, linear calibration curve, and photo images are shown in Figure 8A, 8B, and 8C, respectively. If the concentrations of Ga(III) increased, the absorbance intensity at 620 nm increased and an attendant decrease of the SPR peak at 520 nm was observed. In this regard, the color of Ga(III)-PAH-PVA-Cit-AuNPs solution gradually changed from red to purple and eventually to blue. For quantification of Ga(III), the ratio of the absorbance intensity at 620 nm and 520 nm (A_620_/A_520_) was employed. Based on this, the linear range for Ga(III) was 34.9–418.3 μg/L. The calibration equation constructed was (A_620_/A_520_) = 0.0032 C (μg/L) + 0.0565 with a correlation coefficient (R^2^) of 0.9946, where A was the absorbance value at 620 nm and 520 nm and C was the concentration of Ga(III) (µg/L). Here the limits of detection (LoD) and quantification (LoQ) were calculated according to equations: LoD = 3.3S_b_/m and LoQ = 10S_b_/m where S_b_ is the standard deviation of five measurements of the reagent blank, and m, the slope of the calibration graph. Based on this principle, the estimated LoD and LoQ values were 7.6 μg/mL and 25 μg/mL, respectively. 

### 3.6. Reproducibility and stability

The Col probe reproducibility was inspected by five successive measurements of Ga(III) at PAH-PVA-Cit-AuNPs by spectrophotometry. The suitable relative standard deviation (RSD) of 2.6% obtained, demonstrated the Col probe as satisfactory reproducible. Further, the Col sensor’s stability was analyzed. When the Col sensor material (PVA-Cit-AuNPs) was not in use, it was stored at 4 °C and tested by periodically checking its relative performance. It was measured by storing PVA-Cit-AuNPs for 15 days. After that, the absorbance density almost remained constant with an RSD of 2.9% consequently, confirming the stability and sufficient reproducibility of the Col probe.

### 3.7. Interference studies 

**Figure 9:**
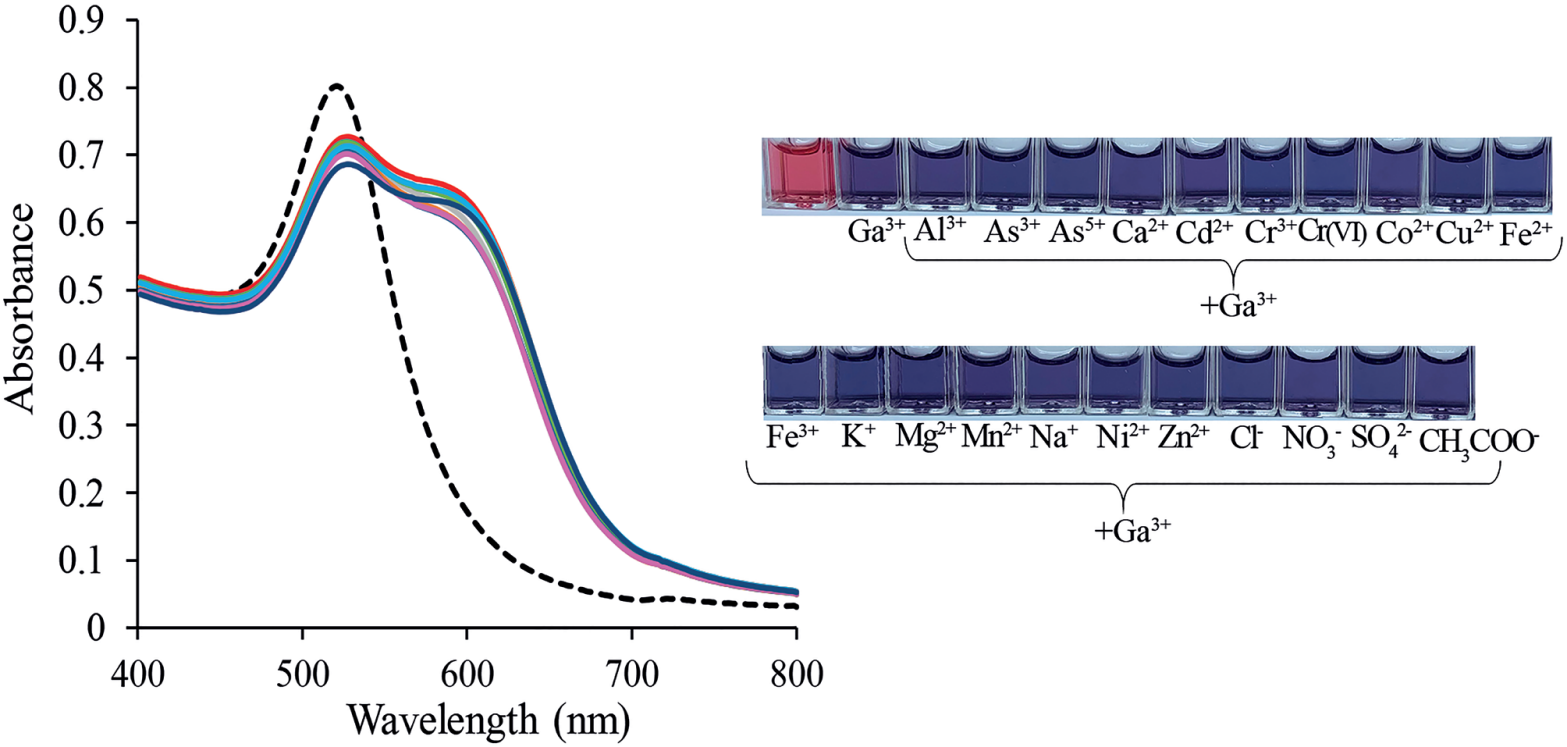
UV-vis absorption spectra (A) and photographs (B) of PAH-PVA-Cit-AuNPs sensor in presence of different foreign ions. [Ga]: 209.2 μg/L.

To evaluate the selectivity of the designed Col sensor for the quantification of Ga(III), the influence of various foreign species on the determination of Ga(III) were evaluated under optimum experimental conditions. Figure 9 shows the UV-vis spectra (A) and photographs (B) of the PAH-PVA-Cit-AuNPs sensor in the presence of different foreign ions. The tolerance limit was taken as the maximum concentration of foreign species causing approximately ±5% relative error in the determination of Ga(III). During interference studies, no masking or complexing agent was utilized. The results provided in Table 1 exhibit that different cations and anions failed to affect the selectivity of the Col sensor. As a result, the PAH-PVA-Cit-AuNPs probe displayed high selectivity for Ga(III) over the other ions tested.

**Table 1:** Interference study of for the determination of 209.2 μg/L Ga(III) under optimum conditions.

Interfering Ion	Added amount(µg/L)	Tolerance limit(Wion/Wanalyte)	Recovery (%)
As3+	75,000	359	99.4
As5+	75,000	359	97.3
K+	39,000	186	99.8
Mn2+	24,750	118	97.2
Na+	23,000	110	101.2
Cd2+	22,400	107	96.6
Mg2+	14,400	69	98.7
Zn2+	13,000	62	101.0
Cu2+	7620	36	98.6
Ni2+	7080	34	96.3
Fe2+	6720	32	99.5
Fe3+	6720	32	97.2
Al3+	6480	31	99.5
Ca2+	6000	29	97.2
Co2+	5400	26	98.6
Cr3+	624	3	101.4
Cr(VI)	624	3	100.6
SO42-	96,000	459	97.8
NO3-	62,000	296	99.2
CH3COO-	59,000	282	100.3
Cl¯	35,500	170	99.8

### 3.8. Comparison with other colorimetric methods

To date, for the spectrophotometric detection and quantification of gallium, 2-(5-bromo-2-pyridylazo)-5-diethylaminophenol (5-Br-PADAP) [41] ,1-(2-benzothiazolylazo)-2-hydroxy-3-naphthoic acid (BTAHN) [42], Chrome Azurol S (CAS) [43], 2-Hydroxynaphthaldehydebenzoylhydrazone (OHNABH) [44], Bipyridylglyoxal bis(4-phenyl-3-thiosemicarbazone (BGPT) [45], 2‐hydroxy‐3‐methoxy benzaldehyde isonicotinoylhydrazone (HMBAINH) [46], rutine [47] and so on have been reported as chromogenic reagents for the determination of Ga(III). A comparison of this suggested work with those of other Ga(III) assay protocols introduced in previous literature is exhibited in Table 2. The linear response range and limit of detection for this developed work were comparable to those declared in the available literature. Compared with other notified chromogenic response based Col sensing system, it exhibits a confident selectivity to Ga(III). Moreover, the concentration level of Ga(III) could be accurately determined with the unaided eye or UV–vis spectrometer. Therefore, the AuNPs-based Col probe can be suitable for instant and on-site monitoring of Ga(III).

**Table 2:** Comparison of analytical parameters of proposed colorimetric sensor with other methods for Ga(III) determination.

Reagent	Linear range(µg/L)	LoD(µg/L)	Referance
5-Br-PADAP	23–700	12	[41]
BTAHN	10–700	3.1	[42]
CAS	20–600	7.6	[43]
OHNABH	10–2000	1000	[44]
BGPT	200–20000	22	[45]
HMBAINH	69–1394	Nr	[46]
Rutine	46–2230	14	[47]
PAH-PVA-Cit-AuNPs	34.9–418.3	7.6	This work

### 3.9. Application of the method

In this study, we selected a tap water sample. Tap water was collected from our lab (İstanbul/Turkey) and used recovery to investigate the application possibility of the Col sensor. Besides, the recommended Col approach was efficiently applied to the detection of trace quantities of Ga(III) in certified reference materials. (TMDA 51.3 fortified water and TMDA 28.3 fortified water) Ga(III) contents of water samples were measured 3 times to obtain precision. For visual detection of Ga(III), a 0.4 mL sample (spiked with gallium) used and analyzed by the developed procedure. It can be seen from Table 3 that the relative standard deviation was in the range of 1.9%–5.9%, and the analytical recovery was between 95.4%–102.0%. All these displayed adequate precision and high accuracy. Hence, the developed Col sensing approach could be employed to the detection of Ga(III) levels in water samples. 

**Table 3:** Tnalytical results and recoveries with real samples by PAH-PVA-Cit-AuNPs probe.

Sample	Added (µg/L)	Found (µg/L)	RSD(%)	Recovery(%)
Tap water	-	˂ LoD	-	-
	34.9	33.3	5.9	95.4
	69.7	67.2	3.7	96.4
	139.4	137.1	1.9	98.3
TMDA 51.3	-	˂ LoD	-	-
	34.9	35.3	5.2	101.1
	69.7	71.1	3.3	102.0
	139.4	141.6	3.1	101.6
TMDA 28.3	-	t LoD	-	-
	34.9	34.1	5.8	97.7
	69.7	67.1	4.3	96.3
	139.4	136.8	2.2	98.1

## 4. Discussion

A new Col method employed for the detection and quantification of Ga(III) in water samples. The Col sensing protocol based on the aggregation of PAH-PVA-Cit-AuNPs which fabricated for the detection of Ga(III). The presence of Ga(III) induced the gathering of PAH-PVA-Cit-AuNPs through cooperative metal–ligand interaction, resulting in a color change from red to blue that is easily observed with the unaided eye or the UV–vis spectrometer. The recommended method reported here enables a highly sensitive Ga measurement even though it is composed of simple and easy operations, needing neither skilled laboratory support nor complex apparatus. This developed probe was linearly responded against the Ga(III) ions over a wide concentration range (34.9–418.3 μg/L) with an acceptable detection limit of 7.6 μg/L with good reproducibility and repeatability. Moreover, the PAH-PVA-Cit-AuNPs-based probe displayed high sensitivity for Ga(III) in aqueous solutions, and the interferences from most of the foreign ions for Ga(III) were ignored; the contents of Ga(III) were in good accordance with the spiked values. In practice, typical Ga(III) recoveries were between 95.4% and 102%. The recovery values indicated that the assay can be utilized for the sensing of Ga(III) in real samples from different fields with high accuracy. The experimental results substantially indicated that the fabricated Col sensor was potentially feasible for Ga(III) ions. This sensing protocol is technically straightforward but very effective, which is mainly because the chelating agent PAH is low cost and commercially available and PAH can also be smoothly coated to the surface of AuNPs by ligand exchange. This field is still required for innovation, especially in employing the exceptional features of AuNPs for metal sensing applications. One such innovation is organising these heavy metal sensing principles into smartphones or similar transferable electronic tools for mobile sensing.

## References

[ref1] (2020). Accumulation of gallium (Ga) and indium (In) in rice grains in Ga- and In-contaminated paddy soils. Environmental Pollution.

[ref2] (2019). Gallium: environmental pollution and health effects. In: Encyclopedia of Environmental Health. Elsevier.

[ref3] (2004). Toxicity of indium arsenide, gallium arsenide, and aluminium gallium arsenide. Toxicology and Applied Pharmacology.

[ref4] (2012). Gallium poisoning: a rare case report. Food and Chemical Toxicology.

[ref5] (2020). Determination of gallium and indium by solution cathode glow discharge as an excitation source for atomic emission spectrometry. Spectrochimica Acta - Part B Atomic Spectroscopy.

[ref6] (2018). Rapid determination of indium in water samples using a portable solution cathode glow discharge-atomic emission spectrometer. Microchemical Journal.

[ref7] (2019). Determination of gallium in water samples by atomic emission spectrometry based on solution cathode glow discharge. Spectrochimica Acta - Part B Atomic Spectroscopy.

[ref8] (2018). A concise guide for the determination of less-studied technology-critical elements (Nb, Ta, Ga. Spectrochimica Acta - Part B Atomic Spectroscopy.

[ref9] (2013). Direct determination of gallium in bauxite employing ICP OES using the reference element technique for interference elimination. Microchemical Journal.

[ref10] (2018). Sequential determination of gallium, indium, and thallium in environmental samples after preconcentration on halloysite nanotubes using ultrasound-assisted dispersive micro solid-phase extraction. New Journal of Chemistry.

[ref11] (2009). Determination of gallium in human urine by supercritical carbon dioxide extraction and graphite furnace atomic absorption spectrometry. Journal of Hazardous Materials.

[ref12] (2015). Determination of trace amounts of Ga(III) by adsorptive stripping voltammetry with in situ plated bismuth film electrode. Talanta.

[ref13] (2015). Adsorptive cathodic stripping voltammetric method for determination of gallium using an in situ plated lead film electrode. Electroanalysis.

[ref14] (2020). A critical review of recent trends, and a future perspective of optical spectroscopy as PAT in biopharmaceutical downstream processing. Analytical and Bioanalytical Chemistry.

[ref15] (2019). Gold nanoparticle-based colorimetric strategies for chemical and biological sensing applications. Nanomaterials.

[ref16] (2017). Bisetty K. A review of gold and silver nanoparticle-based colorimetric sensing assays. Advanced Engineering Materials.

[ref17] (2015). Monolayer protected gold nanoparticles with metal-ion binding sites: Functional systems for chemosensing applications. Chemical Communications.

[ref18] (2020). Importance of gold nanoparticles for detection of toxic heavy metal ions and vital role in biomedical applications. Materials Research Innovations.

[ref19] (2018). Gold nanoparticle-based colorimetric biosensors. Nanoscale.

[ref20] (2017). Cost effective surface functionalization of gold nanoparticles with a mixed DNA and PEG monolayer for nanotechnology applications. RSC Advances.

[ref21] (2017). Gold nanoparticles as efficient sensors in colorimetric detection of toxic metal ions: a review. Sensors and Actuators, B: Chemical.

[ref22] (2017). Peptide-functionalized gold nanoparticles: Versatile biomaterials for diagnostic and therapeutic applications. Biomaterials Science.

[ref23] (2019). The role of ligands in the chemical synthesis and applications of inorganic nanoparticles. Chemical Reviews.

[ref24] (2019). Functionalized gold nanoparticles for sample preparation: a review. Electrophoresis.

[ref25] (2020). Gold nanoparticle: Synthesis, functionalization, enhancement, drug delivery and therapy: a review. Systematic Review Pharmacy.

[ref26] (2020). Polyvinylalcohol-citrate-stabilized gold nanoparticles supported congo red indicator as an optical sensor for selective colorimetric determination of Cr(III) ion. Polyhedron.

[ref27] (2017). Gold nanoparticles functionalized with 2,6-dimercaptopurine for sensitive and selective colorimetric determination of cadmium(II) in food, biological and environmental samples. Analaytical Methods.

[ref28] (2015). Colorimetric detection of Cr3+ using gold nanoparticles functionalized with 4-amino hippuric acid. Journal of Nanoparticle Research.

[ref29] (2021). Solid-phase extraction of Cr(VI) with magnetic melamine-formaldehyde resins, followed by its colorimetric sensing using gold nanoparticles modified with p-amino hippuric acid. Microchemical Jornal.

[ref30] (2019). Colorimetric sensing of Pb2+ ion by using ag nanoparticles in the presence of dithizone. Chemosensors.

[ref31] (2017). A simple and selective colorimetric mercury (II) sensing system based on chitosan stabilized gold nanoparticles and 2,6-pyridinedicarboxylic acid. Materials Science and Engineering: C.

[ref32] (2016). and visual detection of Cr(VI) using gold nanoparticles modified with 1,5-diphenylcarbazide. Microchimica Acta.

[ref33] (1985). Part I - Historical and preparative aspects, morphology and structure. Gold Bulletin.

[ref34] (2004). Integrated nanoparticle-biomolecule hybrid systems: synthesis, properties, and applications. Angewandte Chemie - International Edition.

[ref35] (2009). Instability of cationic gold nanoparticle bioconjugates: The role of citrate ions. Journal of the American Chemical Society.

[ref36] (2016). Surface analysis of gold nanoparticles functionalized with thiol-modified glucose SAMs for biosensor applications. Frontiers in Chemistry.

[ref37] (2013). p-Amino benzenesulfonic acid functionalized gold nanoparticles: synthesis, colorimetric detection of carbaryl and mechanism study by zeta potential assays. Sensors and Actuators, B: Chemical.

[ref38] (2018). Molecular packing of polyvinyl alcohol in PVA-gold nanoparticles composites and its role on thermo-mechanical properties. Polymer Composites.

[ref39] (1997). The X-ray single crystal structure of a gallium citrate complex (NH4)3[Ga(C6H5O7)2]·4H2O. Journal of the American Chemical Society.

[ref40] (1971). Direct titrimetric microdetermination of thallium(I), indium, and gallium(III) separately. I. microdetermination of indium-thallium(I) and indium-gallium(III) in one solution without separation. Microchemical Journal.

[ref41] (2003). Simultaneous determination of gallium and indium with 2-(5-Bromo-2-pyridylazo)-5-diethylaminophenol in cationic micellar medium using derivative spectrophotometry. Analytical Sciences.

[ref42] (2016). Utility of solid phase extraction for UV-visible spectrophotometric determination of gallium in environmental and biological samples. RSC Advances.

[ref43] (2017). A highly sensitive spectrophotometric method for gallium determination with chrome azurol S in the presence of mixed cationic-nonionic surfactants and its application in plant analysis. Communications in Soil Science and Plant Analysis.

[ref44] (2010). A simple spectrophotometric method for the determination of aluminum insome environmental, biological, soil and pharmaceutical samples using 2-hydroxynaphthaldehydebenzoylhydrazone. Eurasian Journal of Analytical Chemistry.

[ref45] (2007). Spectrophotometric determination of gallium(III) as bipyridylglyoxal bis(4-phenyl-3-thiosemicarbazone)derivative. Asian Journal of Chemistry.

[ref46] (2007). Selective second order derivative spectrophotometric method for the determination of gallium(III) in presence of large excess of indium(III). Analytical Letters.

[ref47] (2002). Spectrophotometric determination of gallium(III) with rutin. Analytical Sciences.

